# Stance of numerous leadership styles and their effect on teaching to sustain academic performance at the high school level

**DOI:** 10.1016/j.heliyon.2024.e36438

**Published:** 2024-08-17

**Authors:** Samra Maqbool, Hafiz Muhammad Ihsan Zafeer, Sufyan Maqbool, Pingfei Zeng, Zineb Draissi, Saima Javed

**Affiliations:** College of Education, Zhejiang Normal University, Jinhua, Zhejiang 321004, China

**Keywords:** Leadership styles, Teachers' perception, Sustain academic performance, High school, Pakistan

## Abstract

The present study focused on three leadership, autocratic, democratic, and Laissez-faire, to sustain high school academic performance. To accomplish this, we used quantitative survey method and employed convenient sampling technique to collect data from 358 high school teachers/educators in various regions of Multan, Punjab, Pakistan. Data collection consisted of administering a survey questionnaire that used a five-point Likert Scale. The questionnaire included four variables: one dependent variable, sustained academic Performance, and three independent variables: Autocratic Leadership, Democratic Leadership, and Laissez-faire Leadership. Following data collection, Cronbach's alpha was used to assess the questionnaire's reliability, while the Kolmogorov-Smirnov test was utilized to confirm the normality of the data. Formal statistical analysis included conducting a correlation study to ascertain the association between Autocratic Leadership, Democratic Leadership, and Laissez-faire Leadership with SAP and the impact of each independent variable on the dependent variable. CFA and SEM were conducted using Linear Structural Relations (LISREL) 8.80. These tests were used to identify relationships and differences among the study participants' opinions. The findings indicate that democratic leadership has a highly positive impact, and autocratic leadership has a moderate impact on sustaining academic performance. In contrast, the laissez-faire leadership style has the lowest impact on sustaining academic performance. Based on the study's findings, it is recommended that school teachers/educators should use a combination of democratic and authoritarian leadership styles in their classes to promote cooperation, student participation, ownership in the learning process and leading to their exceptional performance. Furthermore, the findings suggest that schools should actively promote teacher involvement in administrative tasks and decision-making. Ultimately, by integrating the advantages of both types, it is possible to cultivate a comprehensive educational experience that promotes scholarly achievement and equips students with the necessary skills to tackle future problems.

## 1 Introduction

The adequate leadership style for those in the educational sector incorporates handling daily operations and long-term development and growth. Within the ever-changing realm of secondary education, there has been a significant scholarly interest in examining the impact of leadership styles on the educational setting and academic achievements. The significance of leadership practices in educational institutions is widely acknowledged as crucial determinants that can either facilitate or impede the endeavor to achieve academic success [1].

High schools are pivotal in fostering intellectual advancement, facilitating personal growth, and nurturing the acquisition of skills necessary for lifetime learning. The leadership within these institutions assumes a crucial role in influencing the educational experiences of both educators and students. Various leadership styles have evolved as significant paradigms in education, each characterized by its unique philosophy and approach to cultivating an optimal learning environment [2].

*Autocratic leaders* exert significant authority in decision-making processes, frequently depending on their assessments and directions, with limited involvement from educators or other relevant parties. Well-defined hierarchies, rigorous adherence to established protocols, and a primary emphasis on effectiveness and organizational structure characterize this strategy [3]. When managers adopt an autocratic leadership style, they maintain most of the power and make decisions primarily to force the team to comply [4]. The general populace widely dislikes autocratic leaders because they restrict followers from exercising initiative, evaluating their environment, and engaging in self-improvement [5].

Further, according to Ref. [6] when school administrators adopt an authoritarian leadership style, teachers and students find it unpleasant and intimidating, preventing them from reaching their full potential. However, there are instances in which an authoritarian leadership style is appropriate, such as when there is little time and all the knowledge required to solve a problem and the employees are highly driven. An autocratic educational leader may impose rigid rules and procedures, control curriculum selection, decide how to allocate resources, and handle discipline in a high school setting, all without consulting staff members or instructors [7]. This approach may create a more controlled and organized atmosphere. However, it may also make it harder for individuals directly involved in the teaching and learning processes to collaborate and provide their opinions.

The *democratic leadership* style is distinctive in the ever-changing field of educational leadership because shared decision-making, collaborative processes, and an unwavering commitment to diversity mark it [8]. Democratic leaders proactively engage teachers, staff, and students in decision-making. This particular approach places importance on including collective input, fostering collaboration, and establishing agreement [9].

Within the context of high school settings, democratic leadership aims to establish an all-encompassing atmosphere that takes into account a wide range of perspectives in order to shape educational policies and practices [10]. Democratic leadership instills a sense of assurance among staff and teachers, empowering them to fulfill their responsibilities effectively. Moreover, it recognizes and incentivizes innovative instructors and fosters positive change. Additionally, it creates avenues for growth and development for staff and teachers who may encounter setbacks, enabling them to enhance their professional capabilities [11].

The *laissez-faire leadership* style entails granting teachers’ significant autonomy, thereby limiting direct involvement and enabling individuals to exercise independent decision-making [12]. A need for more micromanagement and a dependence on self-motivation and individual initiative distinguishes this leadership style. Under this leadership style, teachers are afforded the autonomy to explore and implement diverse teaching methodologies. The inherent flexibility of this approach has the potential to foster the development of creative and customized instructional strategies that effectively accommodate a wide range of individual learning preferences and styles [13]. It can also be applied to students, fostering autonomy in their educational endeavors. The use of this technique has the potential to augment critical thinking skills and foster self-directed learning, hence exerting a favorable influence on academic achievement. According to Schmid [14], leadership styles are distinct qualities shown by leaders within an organization, and these styles substantially impact the organizational culture. Ketrah, Musa [15] discovered a significant correlation between leadership styles and students' academic achievement in public elementary schools. According to their analysis, the democratic leadership style was the most often used, followed by the laissez-faire and authoritarian forms. Despite many studies undertaken on this phenomenon from numerous perspectives and circumstances, leftovers Anonymous still needs adequate results. The relationship between the three leadership styles and their effect on teaching to sustain academic performance is primarily unexplored in the Pakistani setting [[16], [17], [18], [19]].

## 2 School leadership in Pakistan

The leadership role of a school in Pakistan is considered essential and is frequently discussed about its progress in the education sector. This progress is crucial as it contributes to the development of responsible citizens and empowers individuals to support attaining the National Education Goals [20]. The high sector holds paramount importance as a fundamental sub-sector within education. The foundation of the educational pyramid is upheld by a fundamental element known as the mattress rock. Based on empirical studies undertaken in Pakistan and other developing countries, it has been shown that primary education incurs the most significant social and private costs of return when compared to secondary and university education.

Until recently, the issue of governance and management at the school level in Pakistan has not been given significant attention. Previous educational programs still need to yield substantial improvements in primary and secondary education quality [21]. The UNESCO report (2006) identifies weak governance and management as the critical factor contributing to the substandard quality of education in the public sector. The imperative for enhanced educational leadership becomes increasingly apparent as we endeavor to succeed in our pursuit of transformation. More research is needed to ensure the ability of policymakers and decision-makers to fully comprehend the extent of issues faced by school leaders in Pakistan [22]**.**

Moreover, school leaders play a crucial role in fostering motivation and supporting educators. The use of effective leadership strategies has the potential to foster a conducive work atmosphere that promotes teacher dedication and facilitates professional development. Implementing leadership practices that emphasize providing continuous professional development opportunities for teachers has the potential to enhance instructional practices, curriculum delivery, and, ultimately, academic outcomes [23].

The varied nature of school leadership in Pakistan significantly impacts teaching practices. Effective leadership is pivotal in establishing a conducive and motivating atmosphere for teachers, ensuring alignment with educational policies, fostering community engagement, and ultimately yielding long-term academic success. The formulation of leadership strategies ought to be culturally appropriate, considering the distinct difficulties and opportunities present within the educational framework of Pakistan. The area of study on various leadership stances and how they affect instruction is to maintain high school student's academic performance.

The present study examines the complex association between leadership styles and their impact on teaching that may identify which leadership styles are most effective in promoting and sustaining academic performance in high schools. Different leadership styles, such as Autocratic, democratic, and laissez-faire were examined to understand their impact. Based on the information above, this study aims to address two overarching research questions. 1: what is the effects of Autocratic, Democratic, and laissez-faire leadership styles to Sustain Academic Performance at the high school level? 2: Is there any relationship of leadership styles and Sustain Academic Performance at the high school level?

## 3 Context of theories

The field of literature contains a plethora of material about leadership and the diverse styles employed by leaders within organizations to enhance the performance of their employees. There exist multiple leadership theories and psychological perspectives. A limited number of them are widely recognized. The leadership behavioral theory centers on examining leaders' behaviors and posits that other leaders can emulate these behaviors. This theory called the style hypothesis, posits that leaders are not inherently influential but can develop their leadership abilities through learnable behaviors. This leadership paradigm places significant emphasis on the acts undertaken by the leader [24].

The subsequent category of leadership theory is contingency leadership, often known as situational theory, which emphasizes the contextual factors surrounding a leader. The primary objective of this theory is to examine the situational ramifications resulting from the attainment or lack of achievement. A leader's effectiveness is contingent upon the situational setting in which they operate. The influence of a leader's personality on their effectiveness is relatively minor, with the contextual and situational factors playing a more significant role in determining success. According to this theoretical framework, influential leaders can adapt their approach to varying circumstances. A comprehensive framework in the field of organizational behavior encompasses various theories, such as Hershey and Blanchard's Situational Theory, Evans and House's Path-Goal Theory, and Fiedler's Contingency Theory [25].

Additional leadership theory is the Great Man Theory [26], commonly known as the Trait Theory, which posits that influential leaders possess inherent qualities and are naturally predisposed to leadership. Individuals possess inherent qualities and abilities that contribute to their exceptional aptitude, which are not susceptible to instruction or acquisition. The trait theory posits that individuals in leadership positions are deemed deserving based on their possession of distinctive traits.

Furthermore, educational leaders employ several leadership styles, including Autocratic, Democratic, and Laissez-Faire. The prevalence of the participative leadership idea varies across different types of businesses. This leadership style is commonly referred to as democratic leadership, which advocates for the active participation of employees in the decision-making processes inside their organizations. The leader assumes the role of a facilitator, guiding a discourse and subsequently synthesizing all proposals to choose the optimal course of action [27].

## 4 Literature review

Analyzing literature entails evaluating previous studies critically and extrapolating conclusions from the corpus of knowledge for a given subject. After conducting a comprehensive study of the existing literature, researchers can formulate hypotheses to serve as a framework for their investigations. Evaluate the developed hypotheses and enhance them by incorporating comments received from peers or mentors. It is imperative to provide clarity and coherence in the assertions. Researchers can engage in a comprehensive literature study and formulate hypotheses that serve as a guiding framework for their empirical studies by adhering to the following procedures. The objective is to make novel contributions to the current corpus of knowledge and enhance comprehension within the discipline. The hypotheses were generated based on two research questions.

### 4.1 Autocratic Leadership and sustain academic performance

The impact of an authoritarian leadership style on maintaining success is an intricate and diverse subject. For instance, Chiang, Chen [28] showed that the work environment deteriorates when authoritarian leaders use tactics to control the emotions of their subordinates. Schuh, Zhang [29] showed that subordinates' efforts may be constrained when superiors adopt an authoritarian leadership style. Schuh, Zhang [29] demonstrated a direct correlation between subordinates' discontent with power distance and the adverse effects of directive leadership on performance.

However, in the context of high school education, an authoritarian leadership style can have both positive and negative effects on teaching and academic performance. It is crucial to highlight that the effectiveness of leadership styles can vary based on various circumstances, including school culture, student characteristics, and the overall educational environment [30]. Further, the impact of an autocratic leadership style on high school teaching and academic performance is nuanced and context-dependent. It is critical for developing a positive learning environment to balance structure and discipline with possibilities for instructor autonomy and student interaction. Moreover, study and sophisticated approaches to leadership are required to understand better and optimize the relationship between leadership style and academic achievements in high school settings [31].

A survey conducted by Lessy, Pary [3] found that respondents acknowledged the impact of leadership style on academic success. The effectiveness, job satisfaction, and school environment directly influence students' academic success [32]. Similarly, Pizzolitto, Verna [31] revealed that performance is also negatively impacted by autocratic leadership because they often refrain from engaging in negotiations or seeking input from staff, students, or the community, instead expecting strict compliance with their directives without any objection. In addition, contingent on the context, autocratic leadership styles can positively and negatively influence performance [33,34]. These results showed that an authoritarian leadership style significantly affects teachers' performance in terms of competence at the school level [6]. As indicated in a study that autocrats have little influence the performance of instructors and organizations [35].

### 4.2 Democratic Leadership and sustain academic performance

In an academic context, democratic leadership has several beneficial effects and approaches to maintaining academic achievement [36]. Democratic leadership in schools settings entails a management approach that encourages cooperation, candid communication, and group decision-making among educators, administrators, and students [37]. This leadership style is distinguished by its inclusiveness, regard for various viewpoints, and dedication to cultivating a sense of community within the school. Academic achievement may be sustained and improved in several ways when democratic leadership is present. Facilitate continuous professional development opportunities for educators to augment their competencies in fostering inclusive and democratic classroom environments. Teachers should be encouraged to cooperate on successful teaching strategies and to exchange best practices [38]. However, concerning the influence of democratic leadership on academic performance and instruction, the efficacy of any leadership style can be influenced by several contextual variables [39]. An approach to democratic leadership may succeed or fail depending on the unique dynamics of the school, the community it serves, and the people involved [40].

Evidence suggests that teachers in the decision-making process, including curriculum development, assessment techniques, and school regulations, may result in a more thorough and efficient educational approach [41]. Hoque and Raya [42] indicated that democratic leadership styles are the most practical styles that substantially positively affect the performance of organizations and educators. According Parveen, Quang Bao Tran [43] stated that teachers' productivity was shown to be significantly affected by the principal's democratic leadership style. On the other hands Imhangbe, Okecha [39] the efficacy of educators was found to be positively correlated with a democratic leadership style, and also revealed that teacher performance improves when teachers are empowered to make decisions, consult with one another, and participate alongside chief teachers. The results corroborate the study's assertion that the democratic leadership style was the most regularly adopted.

### 4.3 Laissez-faire *Leadership and sustain academic performance*

Laissez-faire leadership is distinguished by its non-interventionist nature, in which the leader grants minimal direction and empowers groups or individuals to determine the course of action independently [44]. The impacts of laissez-faire leadership on high school academic achievement may be intricate and contingent upon several conditions. According to the positive impact when leaders exert minimal interference, students are afforded the autonomy to investigate creative and innovative methodologies for acquiring knowledge [45]. Further, it may encourage a thirst for knowledge and the development of critical thinking abilities. Laissez-faire leadership is often criticized for its possible lack of direction [43]. Some students may need a distinct direction for clarity and good academic performance. While the laissez-faire leadership style may have both good and negative influences on high school academic achievement [46].

However, previous literature showed that the laissez-faire leadership style had a moderate negative impact on pupils' academic achievement [45]. The study conducted by Breevaart and Zacher [47] and their findings revealed a significant correlation between the leader's experience with laissez-faire leadership and a decline in subordinates' and the organizations overall level of trust. The laissez-faire leadership style has been seen to be used at several educational institutions. In cases when school administrators used a laissez-faire leadership approach, there was a noticeable decrease in the average standard score of schools. The leadership style used by school administrators may be a contributing factor to pupils' poor academic achievements [48]. Abasilim, Gberevbie [49] added that assumed to erode trust in supervisors and organizations, laissez-faire is among the ineffective and detrimental leadership styles. Minor positive association between the laissez-faire leadership style and high school performance [50].

### 4.4 *Relationship between AL, DL, LFL* leaderships and sustain *academic performance*

Depending on the context, the individuals involved, and the particular objectives of the educational institution [51], different leadership styles including autocratic, democratic, and laissez-faire may exert diverse influences on academic performance. Effective leadership in the field of education necessitates the establishment of an atmosphere that cultivates student engagement, motivation, and academic achievement while also recognizing the varied requirements of students [52,53]. Ferdinandi and Kiwonde [54] the study results indicate a substantial and positive correlation between democratic leadership styles and students' academic performance and suggests that democratic leadership styles significantly impact students' academic accomplishment. In the context of the laissez-faire leadership style, the observed association between this style and students' academic performance was statistically insignificant, indicating a modest negative effect that did not reach the significance threshold. Furthermore, it is worth noting that under the autocratic leadership style, the correlation coefficient between the variables was found to be a moderate association. The findings indicate that a majority of educational administrators exhibit a preference for using a democratic leadership approach.

However, in the study of KILIÇ [55] indicates that a significant proportion of school instructors use a democratic leadership style to a greater extent, a moderate degree of Laissez-faire leadership, and a relatively low level of authoritarian leadership within their educational institutions. As per the findings of Owan, Asuquo [56] democratic and laissez-faire leadership styles collectively had a positive impact on the job performance of educators, whereas autocratic leadership has a negative, feeble, or insignificant influence. The findings suggested that combining democratic and laissez-faire leadership styles positively influenced teachers' job performance. Similarly, Oyugi and Gogo [57] established a robust association between democratic leadership and pupils' academic achievement. The corrected R square value of 0.374 indicates that democratic leadership accounts for 37.4 % of the variability in academic achievement. Furthermore, their investigation revealed that authoritarian leadership was responsible for 43.8 % of the observed variations. In contrast, the laissez-faire leadership style accounted for 15.7 % variation.

According to the literature mentioned above, much research has been done on leadership; however, there is an apparent deficiency in that most of these studies were carried out outside of Pakistan, specifically in the vicinity of District Multan high schools, and none of them have shown how three different leadership styles work. Among the successful organizations, autocratic, democratic, and laissez-faire leadership styles are associated with success. Thus, based on the research questions and literature review, the following hypotheses were formulated, which have been shown below as null and alternative hypotheses.

## 5 Study hypotheses

Ho There is no relationship of Autocratic Leadership on teaching to Sustain Academic Performance.
Ha There is statistically significant relationship of Autocratic Leadership on teaching to Sustain Academic Performance.
Ho There is no relationship of Democratic Leadership on teaching to Sustain Academic Performance.
Ha There is statistically significant relationship of Democratic Leadership on teaching to Sustain Academic Performance.
Ho There is no relationship of Laissez-faire Leadership on teaching to Sustain Academic Performance.
Ha There is statistically significant relationship of Laissez-faire Leadership on teaching to Sustain Academic Performance.

## 6 Conceptual framework

**Fig. 1:**
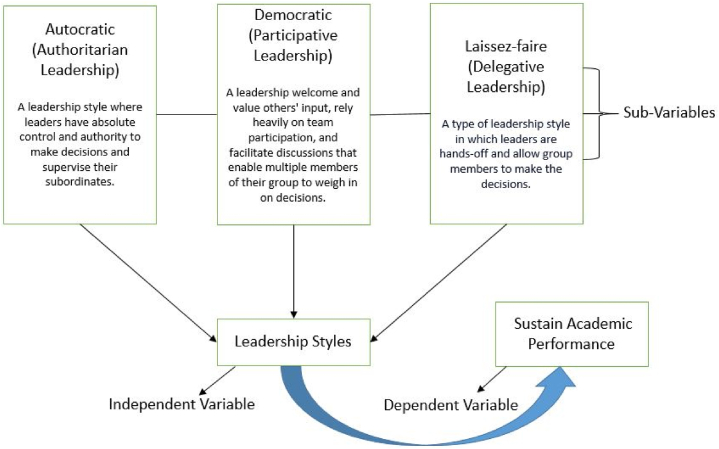
Conceptual representation of present study based on literature review.

The importance of proficient leadership in educational environments is well recognized. The present literature study examines the correlation between leadership styles and academic performance to comprehend the impact of diverse leadership techniques on students' educational achievements. The present conceptual framework depicts the fundamental elements and anticipated connections within the research investigation. It facilitates organizing your inquiry and examining the intricate dynamics between leadership styles and academic achievement, considering variables. Fig. 1 shows the conceptual model of the present study in which we have a dependent variable (sustain academic achievements) and an independent variable (leadership styles). The independent variable is further divided into three sub-variables, namely, autocratic, democratic and laissez-faire leadership, to construct hypotheses based on research question and literature review.

## 7 Methodology

The quantitative survey method was employed [58,59]. The present study includes 358 participants using convenient sampling; 216 females and 142 males comprised the instructors/teachers currently teaching at high schools in Multan, Punjab, Pakistan. The selected schools were under the Board of Intermediate and Secondary Education (BISE). They were between the ages of 26–40 and hold B.A/B.Sc., M.A/M.Sc., and M.Phill degrees along with B.Ed. and M.Ed as professional qualifications. They had between 1 and 11 years of teaching experience.

A survey questionnaire was used to collect the data using leadership styles such as; Autocratic Leadership (AL), Democratic Leadership (DL), Laissez-faire Leadership (LFL), and Sustain Academic Performance (SAP), which consists of four variables one dependent, along with three independent variables. There were a total of 25 items, Autocratic leadership (5 items), Democratic leadership (5 items), Laissez-faire leadership (5 items), and Sustain Academic Performance with 10 items. 5-points Likert scale was used to collect data where 1 represents “strongly disagree,” and 5 represents “strongly agree”. The final questionnaire/instrument was sent to three experts in this field to review to ensure the validity of the questionnaire. In the expert opinion, the questionnaire was valid and appropriate to collect the data.

In 2023, the data-collecting process lasted more than five months. In order to conduct pilot testing, the researcher directly reached out to 70 participants and asked them to complete the questionnaire. Subsequently, Cronbach's alpha was employed to assess the overall reliability of the questionnaire/instruments. Taber [60] stated that Cronbach α values above 0.7 are considered acceptable for research purposes. The overall Cronbach α value of 0.809 obtained in this study indicates a high level of internal consistency, suggesting that the questionnaire is highly reliable for collecting formal data, as supported by the literature review [[61], [62], [63]]. Then, participants were approached at their schools on consented days to collect further data. After finalizing the formal permission, the survey sheets were distributed to participants with a consent letter containing the current research's objectives, details, and guidelines for responding to the questionnaire. It was also assured that their privacy would be kept confidential and that collected information would only be used for academics.

After collecting formal data, the Kolmogorov Smirnov test was employed to ascertain whether the data exhibited a normal distribution [64]. Once the data was found to follow a normal distribution, parametric procedures that were suggested for further data analysis [65]. The study was executed using CFA (Confirmatory Factor study) and SEM (Structural Equation Modelling) using Linear Structural Relations 8.80 (LISREL) [66,67]. Hair, Black [68] emphasized validating all latent variables through CFA. Following that, SEM was utilized. SEM is a reliable multivariate method utilized for testing confirmatory hypotheses [69]. The relationship between observed and latent variables is investigated using the SEM measurement model.

## 8 Findings

The report in Table 1 includes the participants' descriptive and demographic statistics, which include five variables such as; gender, age, educational qualifications, professional qualifications, and experiences, which have been analyzed by frequency, percentage, mean, and standard deviation. Gender (M = 1.60, SD = 0.490), Age (M = 1.71, SD = 0.759), educational qualifications (M = 1.41, SD = 0.581), professional qualifications (M = 1.39, SD = 0.700), and experience (M = 1.90, SD = 0.544).

**Table 1:** Participant demographic statistics.

Variables	**F**	**(%)**	**M**	**SD**
Gender				
Male	142	39.7	1.60	0.490
Female	216	60.3		
Total	358	100.0		
Age				
26–30	170	47.5	1.71	0.759
31–35	122	34.1		
36–40	66	18.4		
Total	358	100.0		
Academic Qualifications				
B.A/B.Sc	229	64.0	1.41	0.581
M.A/M.Sc	112	31.3		
M.phill	17	4.7		
Total	358	100.0		
Professional Qualifications				
B.Ed	265	74.0	1.39	0.700
M.Ed	48	13.4		
Don't Have	45	12.6		
Total	358	100.0		
Experience				
1–5	72	20.1	1.90	0.544
6–10	249	69.6		
11 Above	37	10.3		
Total	358	100.0		

Note: f = frequency, % = percentage, M = mean, SD = standard deviation.

A determination was sought regarding the most suitable statistical analysis by conducting the Kolmogorov–Smirnov test. The significance levels greater than 0.05 are presented in Table 2. The test indicates that parametric statistical approaches are suitable for the given situation, as the data conform to the assumption of normality.

**Table 2:** Results of the Kolmogorov–Smirnov test.

Instruments	Kolmogorov–Smirnov	Sig.(2-tailed)
AL	0.905	0.000
DL	0.947	0.000
LFL	0.933	0.000
SAP	0.936	0.000

Note: AL = autocratic leadership, DL = democratic leadership, LFL = laissez-faire leadership, SAP = sustain academic performance.

Following this, a Pearson product-moment correlation was executed to determine the degree of association and impact of leadership styles like; AL, DL, LFL, and SAP. The impact of Autocratic Leadership (AL), Democratic Leadership (DL), Laissez-faire Leadership (LFL) leadership styles, with Sustain Academic Performance (SAP) was found to be highly positive and statistically significant. Ratner [70] indicated that the r values between 0 and 0.3 were considered weak, while the value between 0 and 0.7 was considered a strong positive impact. Table 3 indicated that H1 Autocratic Leadership (AL) strong (r = 0.863), H2 Democratic Leadership (DL) moderate (r = 0.870) and H3 Laissez-faire Leadership (LFL) have weak (r = 0.528) statistically significant relationship to Sustain Academic Performance SAP. The correlation findings also revealed that AL exhibited a moderate correlation with SAP. At the same time, LFL displayed a comparatively weak correlation with SAP, and DL maintained a strong positive relationship with SAP.

**Table 3:** Measures of correlation between the AL, DL, LFL, and SAP.

Variables	AL	DL	LFL	SAP
	1			
Autocratic Leadership				
	358			
	0.617**	1		
Democratic Leadership	0.000			
	0.678**	0.319**	1	
Laissez-faire Leadership	0.000	0.000		
	0.863**	0.870**	0.528**	1
Sustain Academic Performance	0.000	0.000	0.000	

*Correlation is significant at the 0.01 level (2-tailed).

**Fig. 2:**
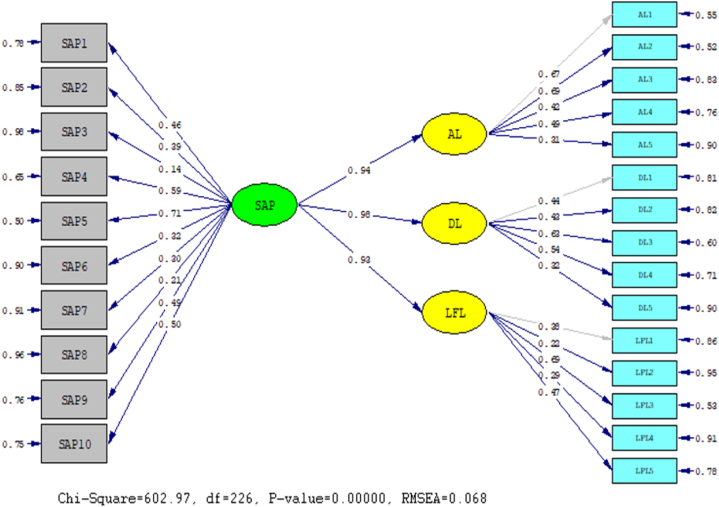
A symbolic representation of the path coefficient values for the relationship between AL, DL, LFL, and SAP.

**Fig. 3:**
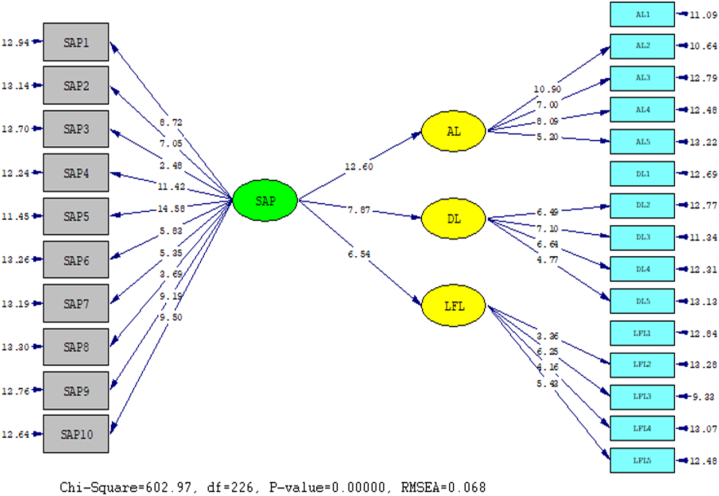
T significance values for path coefficients.

Analyzing the nature of the structural relationship between Autocratic Leadership (AL), Democratic Leadership (DL), Laissez-faire Leadership (LFL) styles and Sustain Academic Performance (SAP), the statistical program LISREL 8.80 was applied in conjunction with CAF and SEM. The outcomes of this evaluation are detailed below: In order to conclude this section, an assessment of the model's fit was conducted through the computation of the chi-square magnitude and the Root Mean Squared Error of Approximation (RMSEA). Each of these indices determines the degree to which the model accurately represents the data (see Fig. 2, Fig. 3).

It provide a summary of the strength of the causal relationships that exist between the variables. Overall result indicated that significantly positive impact of SAP were observed on Autocratic Leadership (AL) (β = 0.94, t = 12.60), Democratic leadership (DL) (β = 0.98, t = 7.87), and laissez-faire leadership (LFL) (β = 0.93, t = 6.54). The results showed that Autocratic Leadership (AL), Democratic leadership (DL) and laissez-faire leadership (LFL) styles statistically highly positive impact on Sustain Academic Performance.

Table 4 displays metrics indicating how well the model fits the data. The RMSEA and the chi-square/df ratio met the acceptable fit criteria.

**Table 4:** Model fit Indices.

Fit Indices	Cut of Score	EM
X^2^		602.97
Df		226
RMSEA	<0.1	0.068

Note: X^2^ = chi-square, df = degree of freedom, RMSEA = Root Mean Square Error of Approximation.

## 9 Discussion

Proficient leadership is critical for the progression of educators as well as society. In the 21st century, the rapid progress of technology has brought forth several issues, one is the need for competent educational leaders to manage educational institutions in the face of global networks of instructors. Improving involvement, communicating a vision, and implementing change are the three pillars upon which the principal's leadership rests when tackling educational and cultural shifts. Education leaders are held in high respect for their ability to raise the bar for education in this era of rapid technological advancement [71,72].

Hallinger [73] stated that leadership styles indirectly impact school performance favorably or adversely. Research has shown that the connection between principals' leadership styles and student success is intricate and complex [74]. The correlation between leadership styles and student accomplishment has been extensively studied for a few decades, and it has shown that leaders possess an indirect but quantifiable impact on students' academic success [75].

This study investigated three leadership styles: authoritarian, democratic, and laissez-faire. The overarching objective was to preserve the academic performance of high school pupils. We constructed a model making use of SEM and CFA, and then we evaluated it. Included in this study were 358 Pakistani high school teachers who had not been the subject of any previous research investigation.

The current study results have shown that democratic, autocratic and laissez-faire leadership styles have a significant relationship and impact on teaching to sustain academic performance, in which democratic leadership (DL) has a strong impact, and autocratic leadership (AL) has a moderating impact. In contrast, laissez-faire leadership (LFL) has a weak impact on teaching to sustain academic performance. However, the involvement of administrators in decision-making, courteous communication with teachers, and efficient work delegation contribute to the improvement of teachers' and school performance.

The results of this research align with Day, Gu [76] findings, which demonstrated that the democratic leadership style of school instructors has a beneficial effect on maintaining students' academic performance, which in turn is directly linked to the success of educational institutions. The results of this research, which were supported by Leithwood, Sun [77] confirmed a favorable correlation between the leadership styles of school instructors and the academic achievement of learners.

Similarly, in a study conducted by Chikoko, Naicker [78] in South Africa, leadership styles have a beneficial impact on the motivation of students to learn. It underscores the need for teachers to adopt a more transformative leadership style since the school's performance is contingent upon the caliber of leadership. They must impart fundamental values to guide their schools, and those values may lead to success effectively. This aligns with the conclusions drawn by Bush [79], who said that there is a strong inclination towards educational leadership due to the prevailing notion that the leadership level substantially impacts both school and student achievements.

Furthermore, Bin Bakr and Alfayez [80] has stated that education enables a nation to attain development and public esteem. The results of this investigation remain consistent with [81]. As indicated by Yalçınkaya, Dağlı [11] instructors' performance is positively influenced by the democratic leadership style of school teachers. Yohannes and Wasonga [13] stated that leadership development is necessary to enhance the effectiveness of educators on a scholastic level. The study findings bring into line with the research carried out by Werang and Lena [82], found that in Indonesian senior high schools, there was a substantial correlation between principal leadership styles and teachers' effectiveness on the job. Concurrently, research done by Saleem, Aslam [83] examined the influence of different leadership styles on teachers' work performance at Pakistan's private schools. The study revealed identical findings within the same setting. Furthermore, the results validated the research conducted by Okoji [84] Within the framework of rural community schools in Nigeria's Ondo State, it was suggested that a combination of democratic and authoritarian leadership styles would enhance the effectiveness of educators in their roles.

As mentioned above, in the present study, it was found that democratic (DL) leadership has a positive impact, and autocratic (AL) leadership has a moderate impact on sustaining academic performance as well as institutions. Similarly, the laissez-faire leadership style of teachers was identified as unhelpful in sustaining students' academic performance. Therefore, in order to enhance teachers' effectiveness and maintain students' academic achievement, it is recommended that school instructors should use a combination of democratic and authoritarian leadership styles. Furthermore, the study's results indicate that schools should actively promote teacher involvement in administrative tasks and decision-making.

## 10 Conclusion and recommendations

The research findings showed that most school instructors demonstrate a predominant use of democratic leadership, moderate authoritarian leadership, and little use of Laissez-faire leadership in their schools. Moreover, studies have shown that when educators embrace a democratic leadership approach, there is a potential for enhanced performance among both students and instructors. The use of democratic and autocratic leadership styles by educators may substantially influence academic achievement, and the efficacy of each style is contingent upon several conditions. Each style has merits and drawbacks, and adopting a well-rounded strategy may provide the most advantageous results. However, teachers need to use democratic leadership to promote cooperation, student participation, and ownership in the learning process. Allowing students to participate in decision-making and problem-solving may instill a sense of empowerment and motivation, leading to their exceptional performance. AL leadership approach may promote critical thinking and engagement among students. Authoritarian leaders have defined goals, a framework, and effective decision-making. Under some circumstances, a directed approach may be essential to uphold discipline and achieve particular educational objectives. Ultimately, a proficient teacher should possess the ability to adapt and use both democratic and autocratic leadership approaches as the circumstances need. Adopting a versatile and context-dependent strategy empowers educators to establish a dynamic learning environment that caters to the varied requirements of learners. By integrating the advantages of both types, it is possible to cultivate a comprehensive educational experience that promotes scholarly achievement and equips students with the necessary skills to tackle future problems.

## Ethical statement

This study was approved by the Ethics Committee of Zhejiang Normal University, Jinhua, Zhejiang, China, with ethics approval reference (ZSRT2024002), and informed consent was also obtained from all participants for this study. The research team adhered rigorously to the university's ethical guidelines and standards during the study to guarantee the responsible and courteous handling of all participants.

## Funding statement

This research did not receive any specific grant from funding agencies in the public, commercial, or not-for-profit sectors.

## Data availability

All the relevant data are included in the manuscript and the supplementary document. No separate repository is attached.

## CRediT authorship contribution statement

**Samra Maqbool:** Writing – original draft, Software, Methodology, Investigation, Formal analysis, Conceptualization. **Hafiz Muhammad Ihsan Zafeer:** Writing – review & editing, Methodology, Formal analysis, Data curation. **Sufyan Maqbool:** Software, Investigation, Data curation. **Pingfei Zeng:** Writing – review & editing, Supervision, Project administration, Funding acquisition. **Zineb Draissi:** Software, Resources, Data curation. **Saima Javed:** Resources, Methodology, Investigation.

## Declaration of competing interest

The authors declare that they have no known competing financial interests or personal relationships that could have appeared to influence the work reported in this paper.
