# Supplementary material for: Stance of numerous leadership styles and their effect on teaching to sustain academic performance at the high school level
Source: Heliyon. 2024 Aug 17;10(16):e36438. doi: 10.1016/j.heliyon.2024.e36438 (PMC11381819; doi:10.1016/j.heliyon.2024.e36438)
Supplement: Multimedia component 1 [file mmc1.docx]

**Appendix**

**Questionnaire**

**Kindly write the necessary information about your profile**

| **Name (optional)**  ***Gender**  ***Age** |  |
| --- | --- |
| ***Academic Qualification:** |  |
| ***Professional Training:** |  |
| ***Experience:** |  |

| **No** | **Autocratic Style** | **SD** | **DA** | **N** | **SA** | **A** |
| --- | --- | --- | --- | --- | --- | --- |
| 1 | I feel my head considers me a subordinate. |  |  |  |  |  |
| 2 | I feel my head never involves me in the decision-making process. |  |  |  |  |  |
| 3 | I feel my head uses hard rules and regulations in school. |  |  |  |  |  |
| 4 | I feel my head discouraged teachers' suggestions in school matters. |  |  |  |  |  |
| 5 | I feel my head implement their own ideas in school. |  |  |  |  |  |
|  | **Democratic Style** |  |  |  |  |  |
| 6 | I feel my head considers me as a team member. |  |  |  |  |  |
| 7 | I feel my head involved me in the decision-making process. |  |  |  |  |  |
| 8 | I feel my head provide me guidance without any pressure. |  |  |  |  |  |
| 9 | I feel my head showed supportive communication with me. |  |  |  |  |  |
| 10 | I feel my head help teachers find their "passion". |  |  |  |  |  |
|  | **Laissez-Faire Style** |  |  |  |  |  |
| 11 | I feel my head order to solve the problem at my ends. |  |  |  |  |  |
| 12 | I feel my head never appraises my work/performance. |  |  |  |  |  |
| 13 | I feel my head never gives me the freedom to solve the problem at my ends. |  |  |  |  |  |
| 14 | I feel my head provides little input toward my problems. |  |  |  |  |  |
| 15 | Generally, my head leaves me alone to complete my job and make decision. |  |  |  |  |  |
|  | **Academic Performance of Students** |  |  |  |  |  |
| 16 | The autocratic leadership style of the teachers improved the academic performance of students. |  |  |  |  |  |
| 17 | The democratic leadership style of the teachers enhanced the academic performance of students. |  |  |  |  |  |
| 18 | The Laissez-faire leadership style of the teachers increased the academic performance of students. |  |  |  |  |  |
| 19 | I feel my problem-solving skills have been improved. |  |  |  |  |  |
| 20 | I feel my communication skills have been improved. |  |  |  |  |  |
| 21 | I feel my teaching skills have been increased. |  |  |  |  |  |
| 22 | I feel my analytical skills have been developed. |  |  |  |  |  |
| 23 | I feel my motivational abilities have been improved. |  |  |  |  |  |
| 24 | I feel my negotiation skills have been improved. |  |  |  |  |  |
| 25 | I feel my research skills have been improved. |  |  |  |  |  |
| 26 | I feel my lesson planning skills have been improved. |  |  |  |  |  |
| 27 | I feel my lesson delivery skills have been increased. |  |  |  |  |  |
| 28 | I can use various aids in my teaching and learning. |  |  |  |  |  |
| 29 | I feel that head encouragement increased my performance. |  |  |  |  |  |
| 30 | I feel that the appreciation of school head enhanced my performance. |  |  |  |  |  |
